# An Ultrathin, Triple-Band Metamaterial Absorber with Wide-Incident-Angle Stability for Conformal Applications at X and Ku Frequency Band

**DOI:** 10.1186/s11671-020-03448-0

**Published:** 2020-11-18

**Authors:** Guangsheng Deng, Kun Lv, Hanxiao Sun, Jun Yang, Zhiping Yin, Ying Li, Baihong Chi, Xiangxiang Li

**Affiliations:** 1 Special Display and Imaging Technology Innovation Center of Anhui Province, Academy of Opto-Electronic Technology, Hefei University of Technology, Hefei, 230009 China; 2 Process and Mechanical Engineering Technology Laboratory, Space Star Technology Co. Ltd., Beijing, 100095 China; 3 723 Research Institute of China Shipbuilding Industry Corporation, Yangzhou, 225101 China

**Keywords:** Metamaterial, Absorber, Conformal, Ultrathin, Multi-band

## Abstract

An ultrathin and flexible metamaterial absorber (MA) with triple absorption peaks is presented in this paper. The proposed absorber has been designed in such a way that three absorption peaks are located at 8.5, 13.5, and 17 GHz (X and Ku bands) with absorption of 99.9%, 99.5%, and 99.9%, respectively. The proposed structure is only 0.4 mm thick, which is approximately 1/88, 1/55, and 1/44 for the respective free space wavelengths of absorption frequency in various bands. The MA is also insensitive due to its symmetric geometry. In addition, the proposed structure exhibits minimum 86% absorption (TE incidence) within 60° angle of incidence. For TM incidence, the proposed absorber exhibits more than 99% absorptivity up to 60° incidence. Surface current and electric field distributions were investigated to analyze the mechanism governing absorption. Parameter analyses were performed for absorption optimization. Moreover, the performance of the MA was experimentally demonstrated in free space on a sample under test with 20 × 30 unit cells fabricated on a flexible dielectric. Under normal incidence, the fabricated MA exhibits near perfect absorption at each absorption peak for all polarization angles, and the experimental results were found to be consistent with simulation results. Due to its advantages of high-efficiency absorption over a broad range of incidence angles, the proposed absorber can be used in energy harvesting and electromagnetic shielding.

## Introduction

In recent years, metamaterials have received widespread concern due to their exotic properties, such as negative refractive index [1], perfect imaging [2], and inverse Doppler effects [3]. Because of these properties, metamaterials have been proposed for use in various devices, such as electromagnetic (EM) cloaking [4], ultra-sensitive sensing [5], filters [6, 7], and absorbers [8–12]. In particular, metamaterial absorbers (MAs), compared with traditional microwave absorbers, are used in a variety of fields, ranging from military to consumer electronics. MAs tend to be lightweight and thin.

In 2008, a perfect MA was first presented by Landy et al. [13]. Subsequently, different types of MAs, such as single-band [14, 15], dual-band [16–21], multi-band [22–27], and wideband absorbers [28–36], have been presented by various researchers. Among these MAs, multi-band MAs enable perfect absorption at several discrete frequencies, enabling applications like multiband sensing. In general, a multi-band MA can be configured with two methods. The first method is commonly known as the coplanar construction method, where several resonators of different sizes are formed into a super-unit structure [37, 38]. The second method involves vertical stacking of alternating multi-layer structures [39, 40]. However, neither of these methods is ideal for fabricating a structure that provides multiband absorption. For example, coplanar construction method leads to an inevitably expanding of the MA unit size, whereas the layered design could not eliminate the disadvantage of large thickness and heavy weight of the structure. Recently, some simplified structural designs were presented to achieve multi-band absorption [41, 42]; nevertheless, the absorption at wide incident angle still needs to be improved.

In this paper, we propose a design method that combines the advantage of compact-size, ultra-thin, light-weight, and easy-to-fabricate. In merit of the unit cell design, the proposed triple-band MA exhibits high absorption even at wide angles of incidence. Simulation results reveal three distinct absorption bands with peak absorption of 99.9%, 99.5%, and 99.9% at 8.5, 13.5, and 17 GHz, respectively. The symmetric structure of the MA ensures its absorption is insensitive to different polarization angles. Moreover, the proposed MA offers absorption greater than 86% and 99% when TE and TM-polarized waves are incident at 60° angle of incidence, respectively. The relationship between various geometric parameters and the absorption spectrum was examined. To validate the absorbing performance of the MA, a prototype with 20 × 30 unit cells was fabricated, and the experimental results are found to be consistent with simulation results. Due to its low thickness and effectiveness for a broad range of incident angles, the MA structure was fabricated on a highly flexible polyimide film, which can be used in non-planar and conformal applications.

## Methods/Experimental

**Fig. 1:**
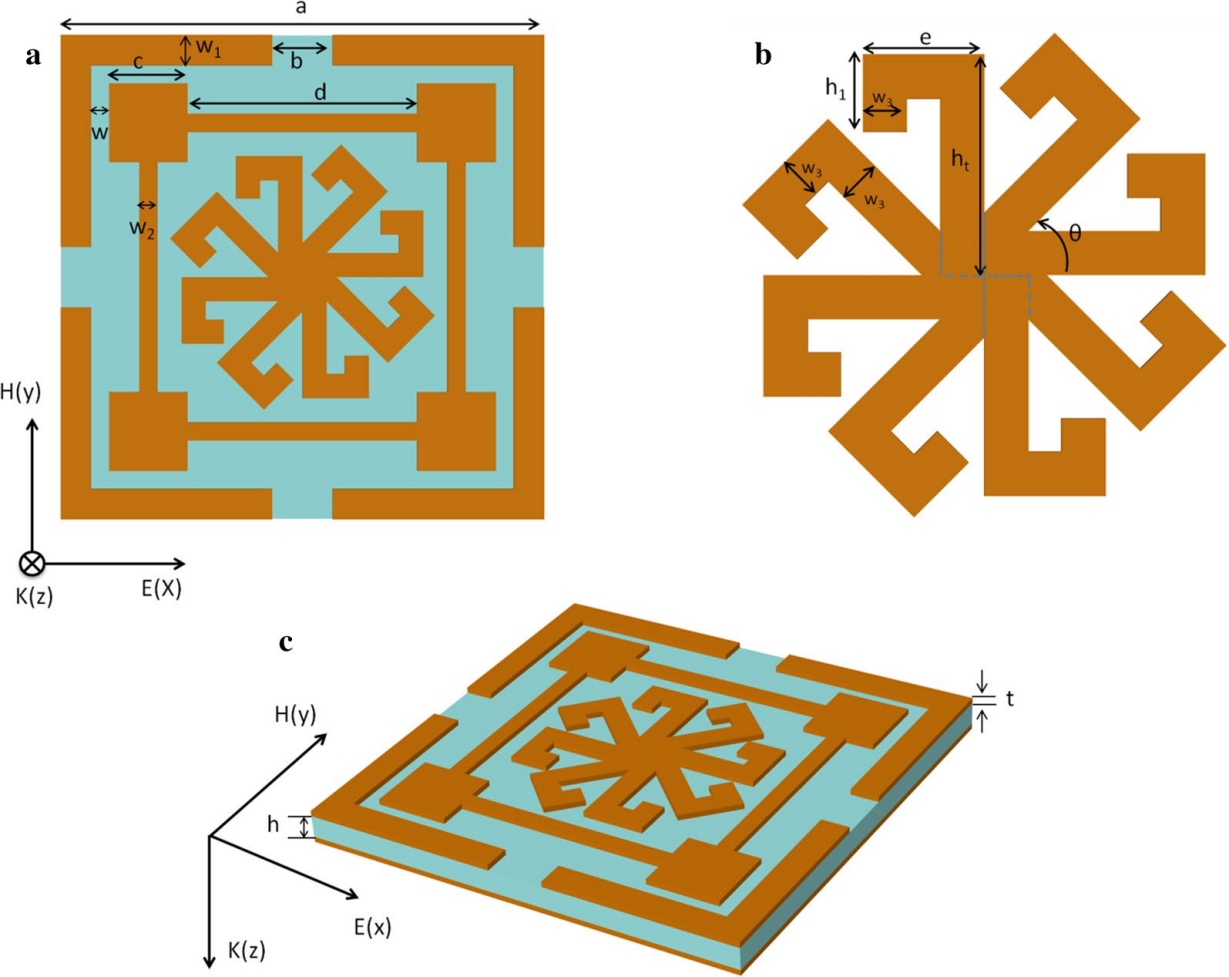
Schematic geometry of a unit cell for the proposed MA. **a** Top view, **b** layout of the eight 7-shaped resonance structures, and **c** perspective view of a unit cell

Figure 1 shows the geometry of the unit cell for the proposed MA, which consists of a resonance layer, a dielectric layer, and a metallic ground layer. The resonant structure combines a split ring resonator (SRR), a modified ring resonator (MRR), and eight identical 7-shaped structures, each rotated 45° along the unit' center. The top patterned layer and bottom ground layer are made of 0.02-mm-thick copper and an electric conductively of 5.8 × 10^7^ S/m. The substrate was fabricated on polyimide with relative permittivity of 2.9 and loss tangent of 0.02. The optimized parameters of the MA are listed in Table 1.

**Table 1:** Dimensions and parameters of the MA

Parameter	Value (mm)	Parameter	Value (mm)
*a*	8	*w*_2_	0.3
*b*	1	*w*_3_	0.5
*c*	1.3	*h*	0.4
*d*	3.8	*h*_1_	0.7
*e*	1.2	*h*_t_	2
*w*	0.3	*t*	0.02
*w*_1_	0.5	*θ*	45°

**Fig. 2:**
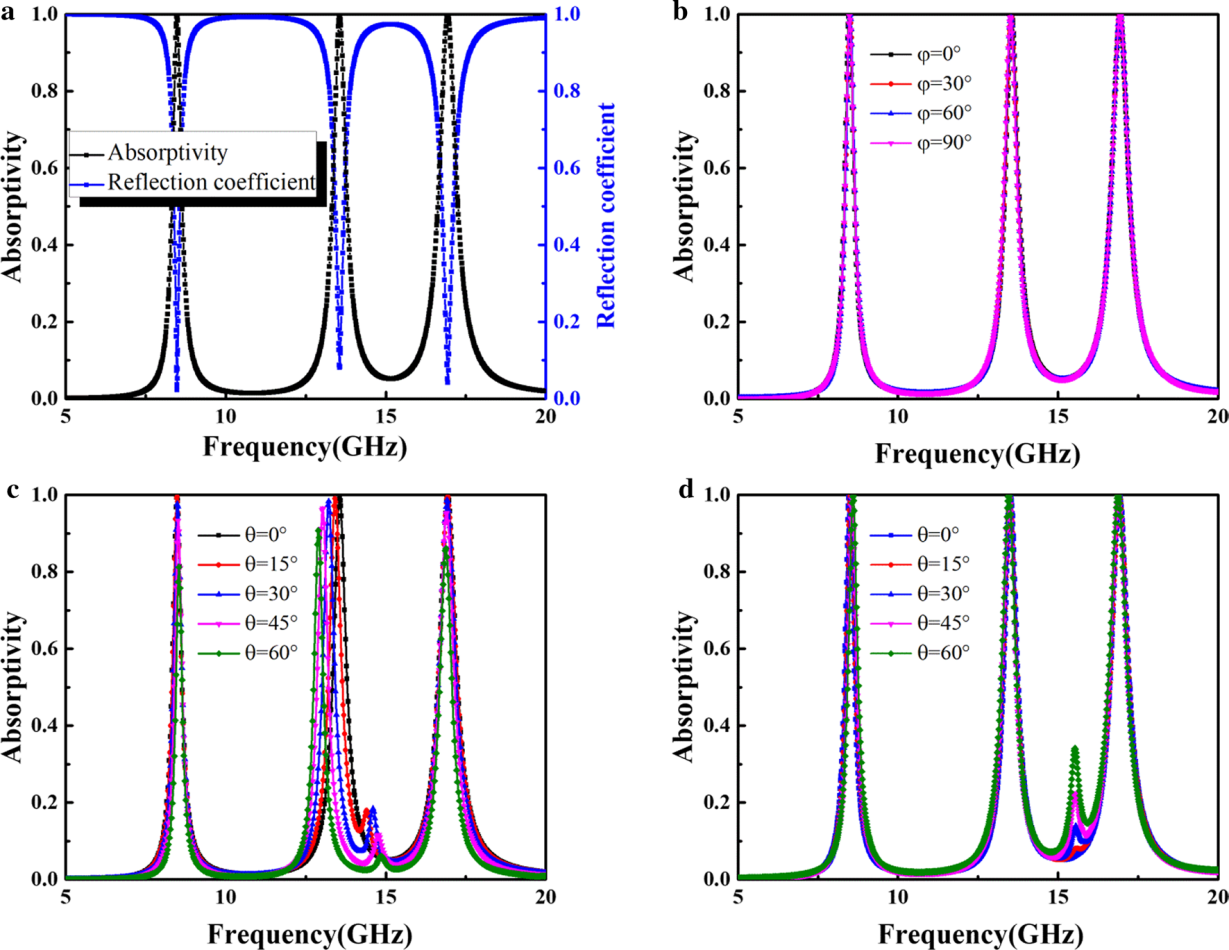
**a** Simulated absorption and reflection spectra at normal incidence. **b** Absorption spectrum for different polarization angles *φ*. Absorption for different *θ* values for **c** TE and **d** TM polarization

The simulated absorption spectra of the proposed MA were determined from a finite-difference time-domain (FDTD) simulation. In the simulation, the unit cell boundary conditions were applied in the x and y directions, while the Floquet port condition was imposed along the z direction. Moreover, a plane EM wave was assumed to hits the surface of the MA. The absorptivity (*A*) can be defined as $A\left(\upomega \right)=1-{|{S}_{11}(\upomega )|}^{2}-{|{S}_{21}(\upomega )|}^{2}$, where ${S}_{11}(\upomega )$ and ${S}_{21}(\upomega )$ are the reflection and the transmission coefficients, respectively. Since the transmission coefficient ${S}_{21}(\upomega )$ is zero due to the total reflection of copper ground plane, the absorptivity can be simplified as $A\left(\upomega \right)=1-{|{S}_{11}(\upomega )|}^{2}$. The simulated reflection and absorption spectra of the proposed MA under normal incidence are shown in Fig. 2a. The proposed MA exhibits three absorption peaks at 8.5, 13.5, and 17 GHz with absorption of 99.9%, 99.5%, and 99.9%, respectively; the corresponding *Q* factor of each resonant mode can reach 26.8, 28.4, and 27.1, respectively.

Figure 2b shows the absorption spectra of the proposed MA structure for different polarization angles. One can see that the absorption of the MA remains stable for polarization angle ranging from 0° to 90°. Therefore, the proposed MA is insensitive to polarization of incident EM waves. In addition, we further investigated absorption in the designed MA at oblique angle of incidence (*θ*). For TE polarization, as shown in Fig. 2c, the absorptivity decreases as *θ* increases. This may occur because increasing *θ* decreases the horizontal component of the electric field intensity for TE waves. Therefore, the effectiveness of the circulating current generated by the incident electric field gradually decreases. However, the three absorption peaks remain above 86% as *θ* reach up to 60°. For TM polarization, as shown in Fig. 2d, the absorptivity at each resonance peak is greater than 99% at *θ* = 60°. This occurs because absorption in the proposed MA is less sensitive to changes in the electric field intensity caused by an increase in *θ*. Another advantage of the proposed MA is the absorption frequency stability, as shown in Fig. 2, where the three distinct absorption peaks do not change significantly as *θ* increases.

## Results and Discussion

**Fig. 3:**
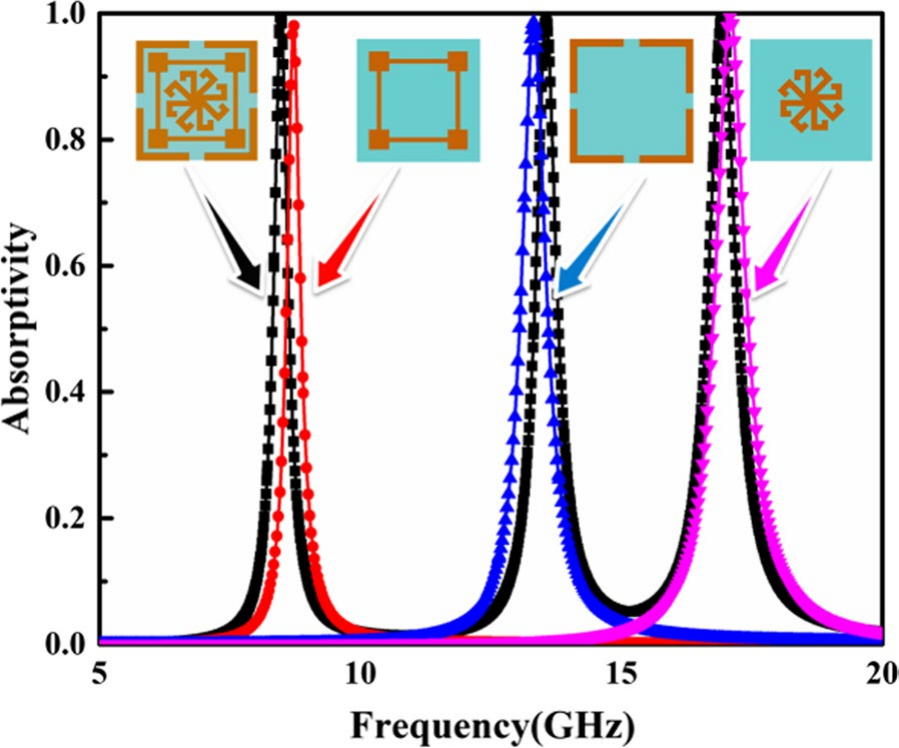
Contribution from individual elements to absorption

In order to facilitate a detailed explanation of absorption, the response spectra for different parts of the resonance structure are presented in Fig. 3. As shown in Fig. 3, each element within the patterned layer is responsible for an individual and intense resonance. As a result, a combination of these elements leads to perfect multiband absorption. As part of the MRR design, a square patch is added to each corner of the closed ring resonator, which increases the electrical length of the ring resonator and red-shifts the absorption frequency without increasing the size of the structure.

**Fig. 4:**
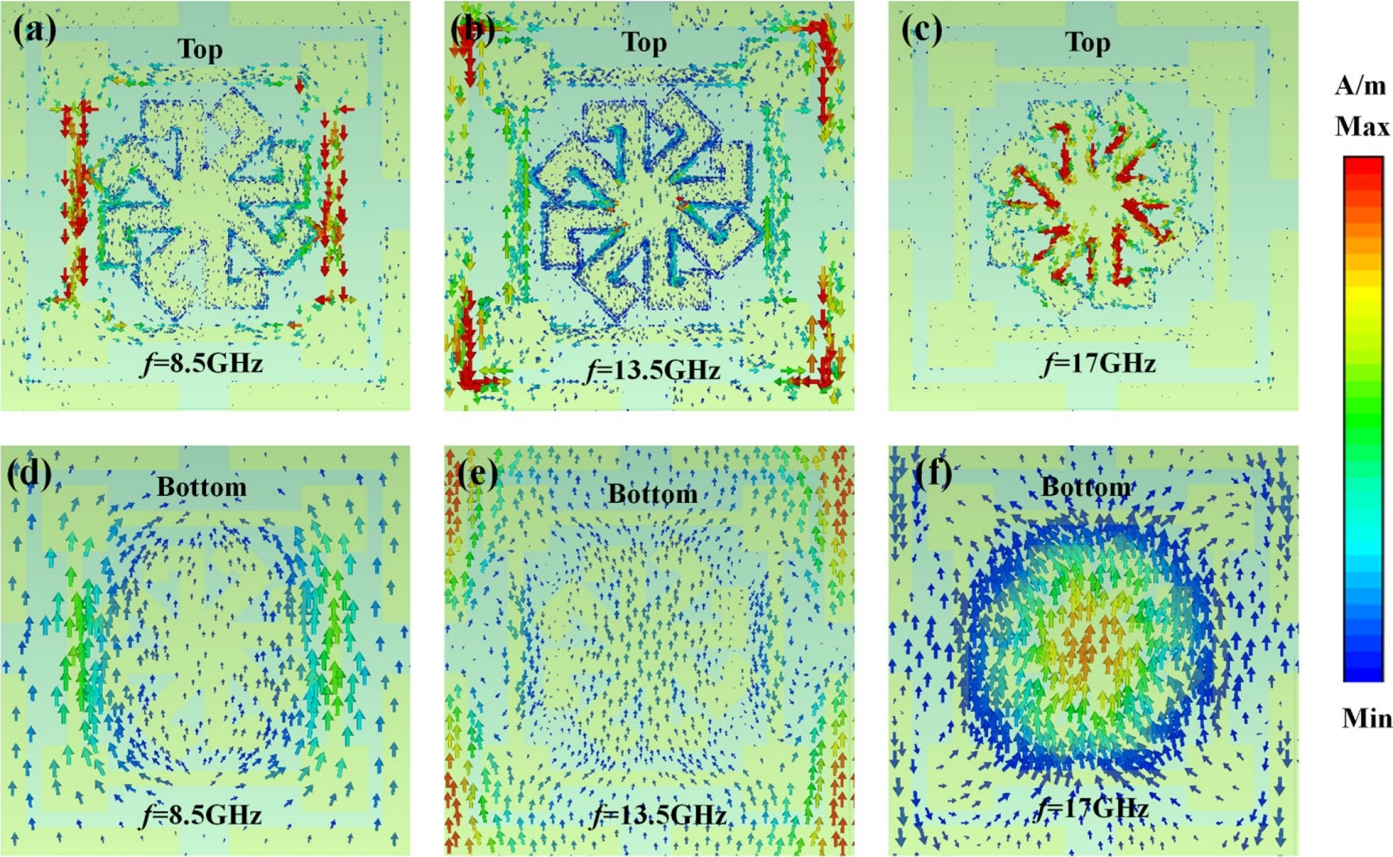
Simulated surface current distribution on the top patterned layer and bottom ground layer at **a**, **d** 8.5, **b**, **e** 13.5, and **c**, **f** 17 GHz

**Fig. 5:**
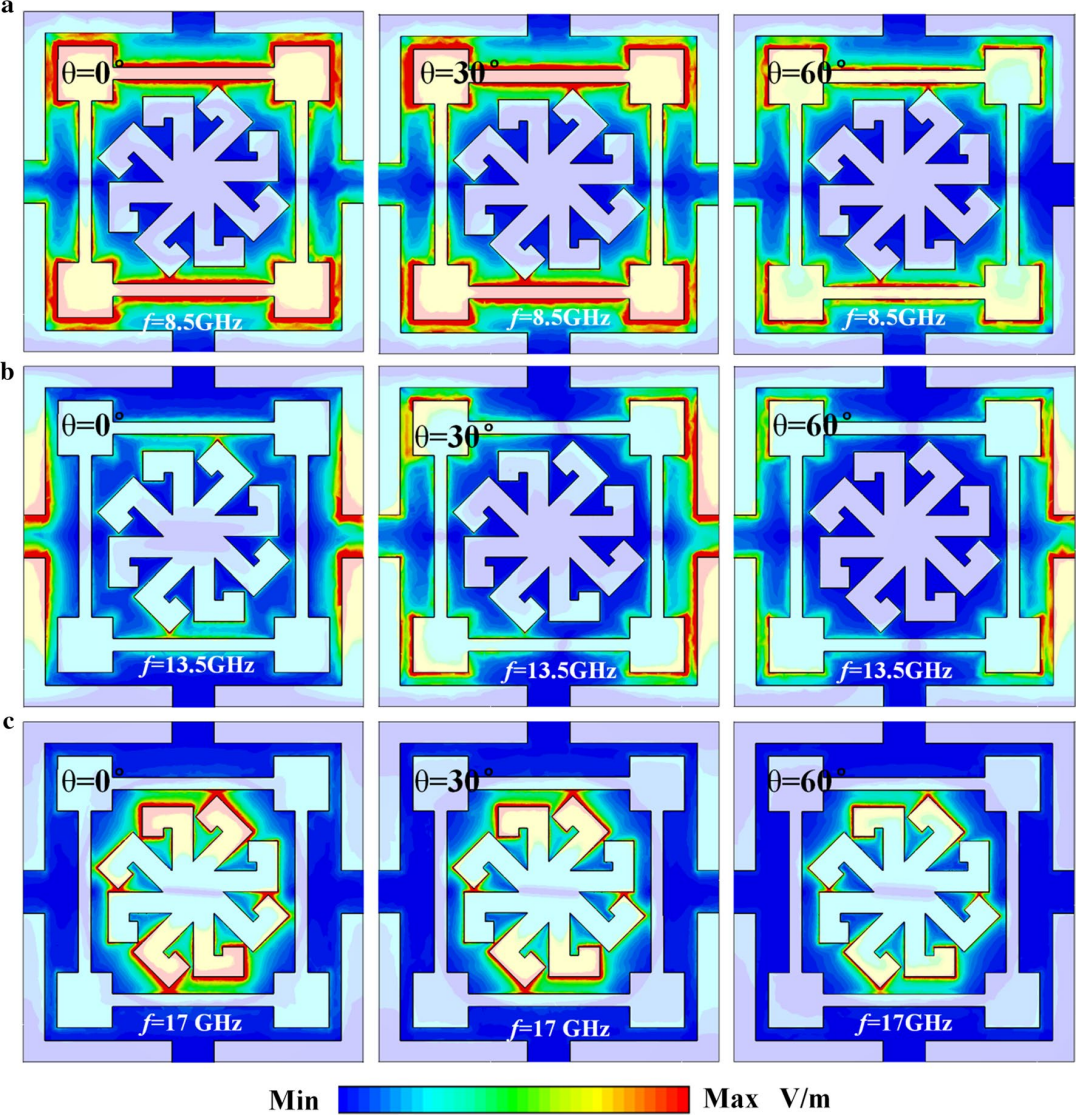
The absolute of electric field distribution (|*E*|) in the MA for TE polarization at different incident angle *θ* of **a** 8.5, **b** 13.5, and **c** 17 GHz

To further explore the mechanism of EM wave absorption, surface current density distributions on the top and bottom metallic layers corresponding to three absorption peaks are shown in Fig. 4. One can see that the surface current on the top patterned layer is concentrated on the MRR, the SRR, and the 7-shaped graphic structures at 8.5, 13.5, and 17 GHz, respectively. The surface current distribution also reveals the origin of wave absorption, as shown in Fig. 3. Compared with the surface current on the top layer, the intensity on the bottom ground layer is much weaker. The direction of the surface current on top layer is anti-parallel with respect to ground plane, which results in equivalent current loops within the MA that excites a magnetic dipole. Meanwhile, Fig. 5 shows the amplitude of the electric field (|*E*|) in the MA for incident TE-polarization waves when *θ* = 0°, 30°, and 60°. One can see that the electric field is strongly concentrated on the horizontal bars of the MRR as the MRR absorbs at 8.5 GHz. At 13.5 GHz, as shown in Fig. 5(b), perfect absorption is due to the LC resonance in the SRR. Finally, absorption at 17 GHz is due to a dipole resonance in the inner patch. The resonators in the top layer also develop electric resonances. Both the magnetic and electric resonances contribute to strong EM absorption in the proposed structure. In addition, Fig. 5 shows that the field intensity decrease as *θ* increases. As a result, EM wave absorption also decreases with the increase of *θ*.

**Fig. 6:**
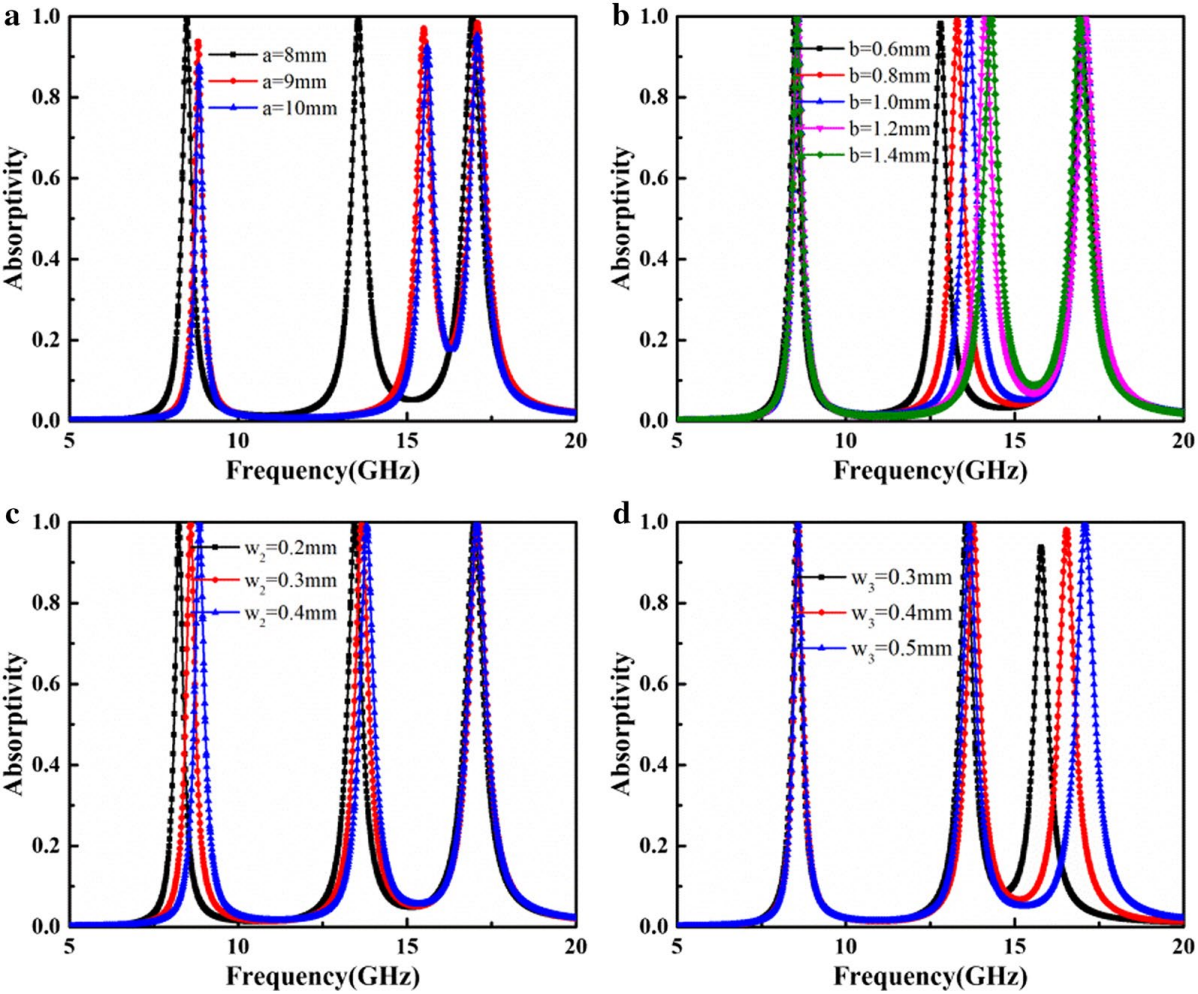
Absorption spectrum of the MA for different structural parameters: **a** unit periodicity *a*, **b** SRR gap width *b*, **c** MSR ring bar width *w*_2_, **d** 7-shaped patch width *w*_3_

Figure 6 shows the effects of the MA geometry on absorption in the proposed MA. As shown in Fig. 6a, the resonant frequencies shift toward higher frequencies as *a* increases. The relationship between gap width *b* of the SRR and the absorption spectrum is shown in Fig. 6b. The equivalent capacitance decreases as b increases; thus, the center resonant peak shifts to higher frequencies. However, the lower and upper absorption peaks remain almost unchanged, which provides a convenient way to tune individual absorption frequencies. Moreover, the dependence of absorption on the width of the ring bar *w*_2_ is presented in Fig. 6c, where both the lower and center resonant frequencies red-shift as *w*_2_ increases. As *w*_2_ increases, the equivalent capacitance increases because the distance between the SRR and MRR decreases, causing the lower and center resonant frequencies to red-shift. Finally, increasing the bar width *w*_3_ will cause a red-shift in the upper resonant frequency, as shown in Fig. 6d. As the resonant mode is determined by the inner 7-shaped patch, increasing *w*_3_ also increases the equivalent inductance of the inner resonator. Therefore, the resonant frequency exhibits a red-shift.

**Fig. 7:**
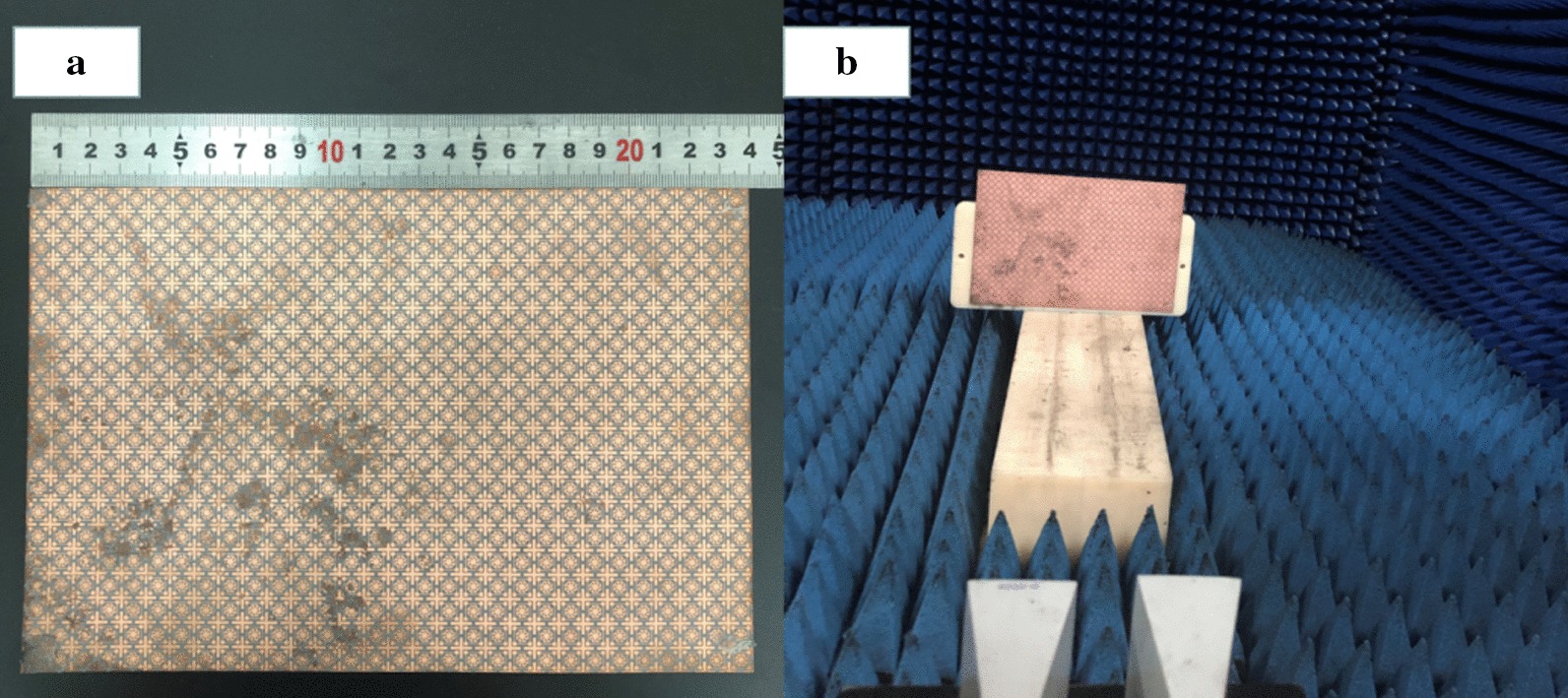
**a** Fabricated MA prototype. **b** Measurement setup

**Fig. 8:**
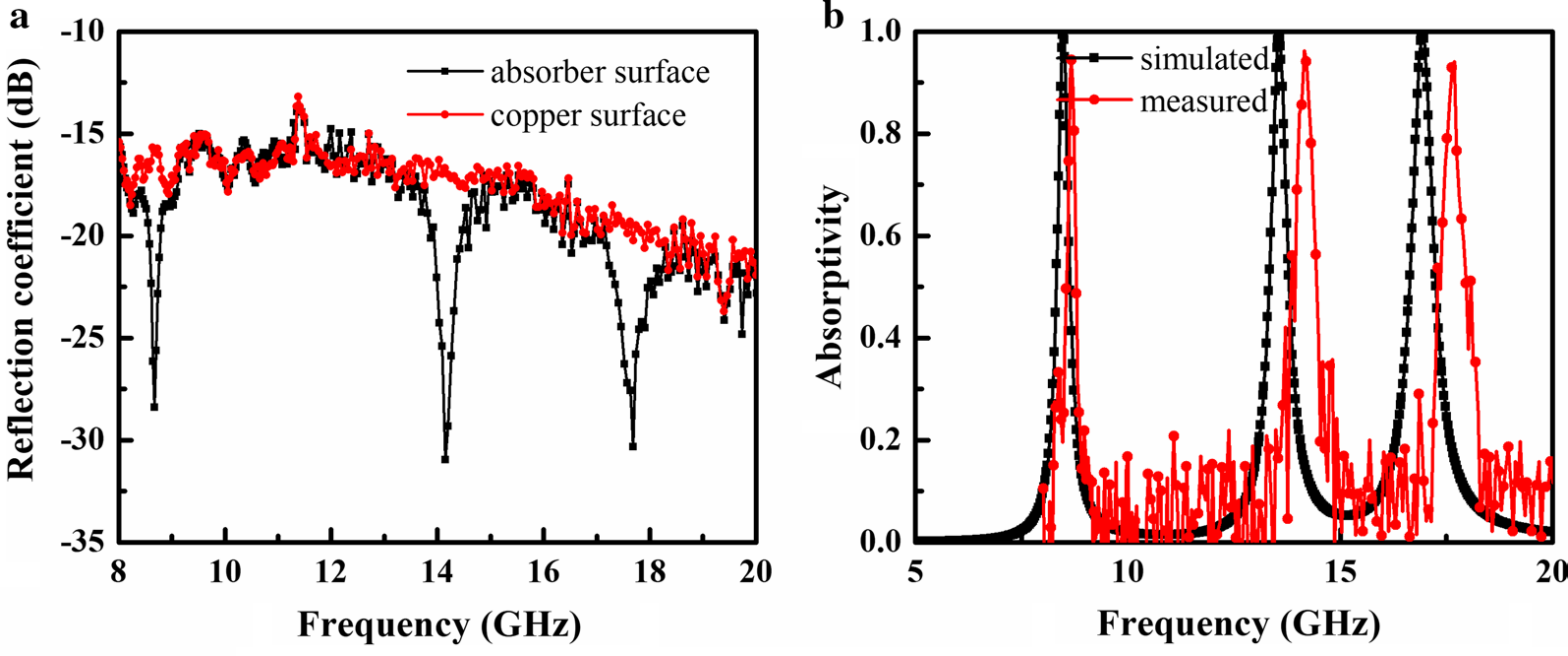
**a** Reflection coefficient and **b** absorptivity of the MA at normal incidence

A 240 mm × 160 mm prototype, corresponding to 20 × 30 unit cells, was fabricated, as shown in Fig. 7a. In the sample preparation, a thin layer of copper was evaporated on the surface of the polyimide, and then the patterns were etched using laser ablation. The measurement setup is shown in Fig. 7b, where absorption in the sample was tested with the free space method. A pair of horn antennas was connected to a vector network analyzer (Rohde & Schwarz ZVA 40) to measure reflection from the sample. The reflection spectrum for a copper plate with the same size as the fabricated sample was measured and used as a reference. The sample was then placed at the same location and the real reflection from the sample was calculated by subtracting the two measured reflected powers. Figure 8a shows the reflection spectrum measured from the copper plate and the fabricated sample, while the absorptivity of the MA is shown in Fig. 8b. The measured absorption is 96%, 97%, and 94% at 8.7, 14.1, and 17.6 GHz, respectively. Compared with the simulation results, the absorption peak frequencies move slightly toward higher frequencies due to manufacturing tolerances and differences in the substrate’s permittivity.

**Fig. 9:**
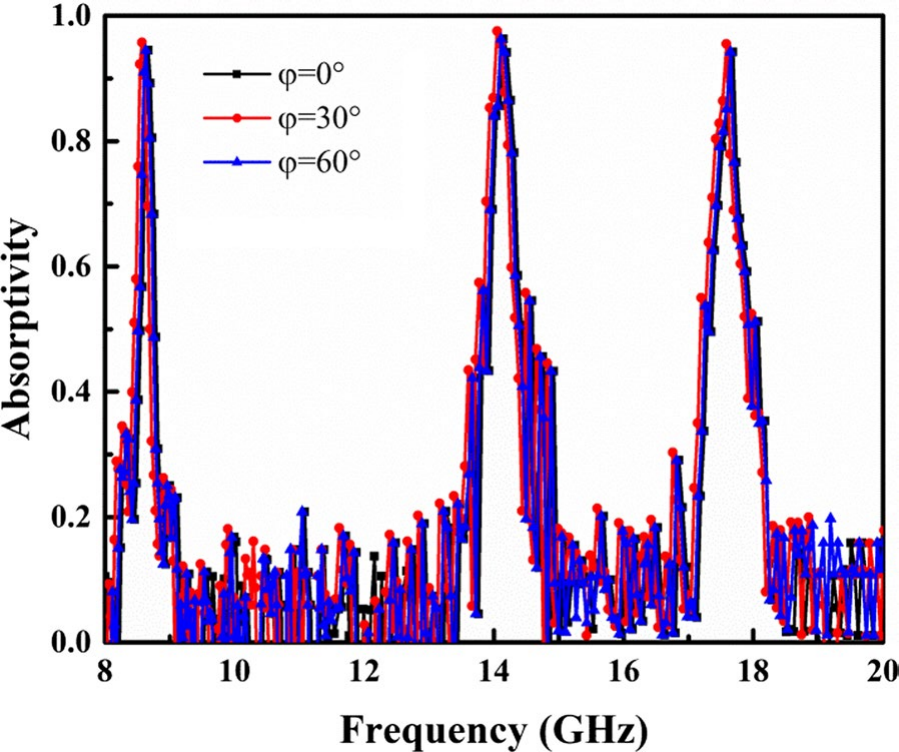
Measured absorption for different polarization angles under normal incidence

**Fig. 10:**
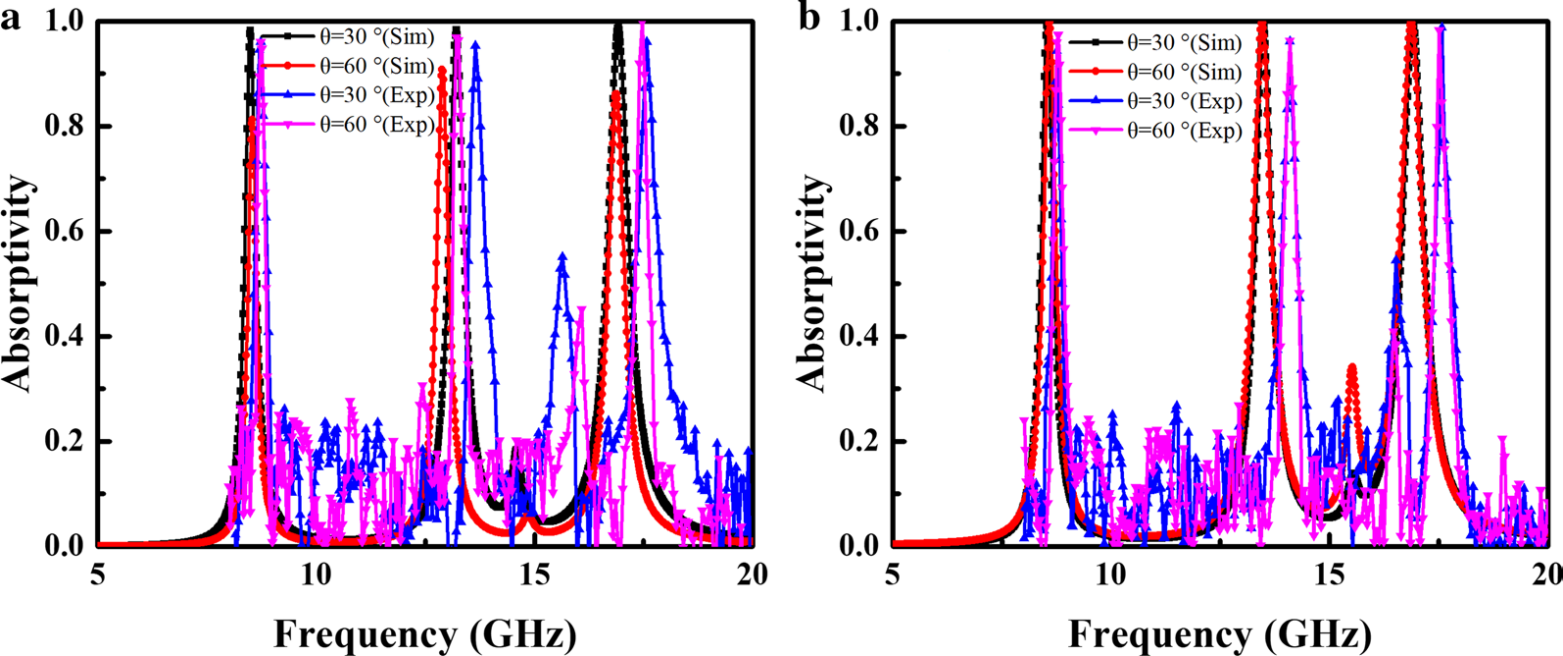
Simulated and measured absorption for different incident angles: **a** TE and **b** TM polarization

Figure 9 shows absorption in the MA measured at different polarization angles of *φ* = 0°, 30°, and 60°. The result shows that the proposed structure is insensitive to polarization angle. Figure 10 shows the measured absorption spectra for TE and TM polarization when *θ* = 30° and 60°. Absorption for both polarizations remains above 95% when *θ* = 60° for all absorption peaks.

**Fig. 11:**
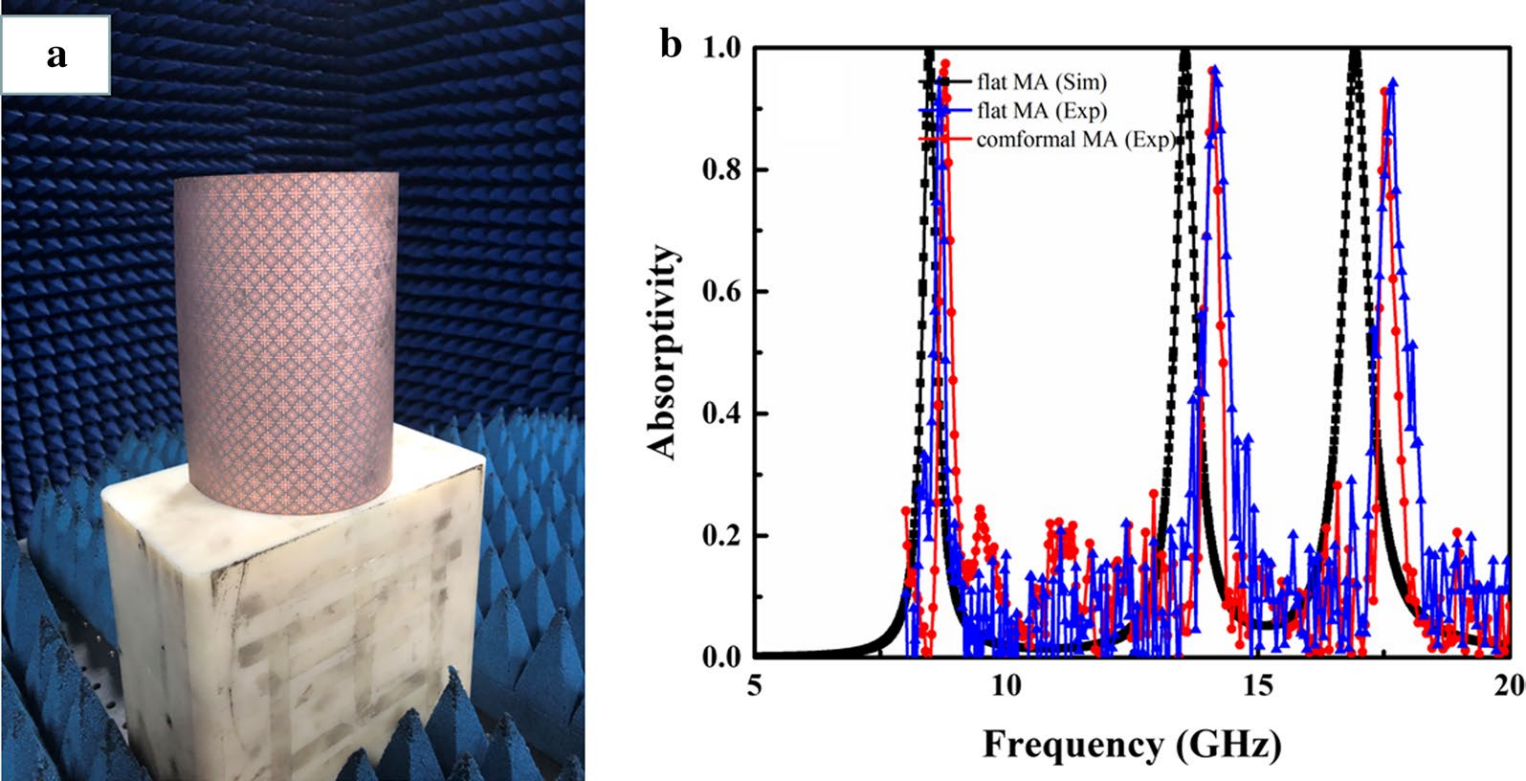
**a** Flexible absorber attached to a cylinder. **b** Absorption spectra of the flat and conformal MA

As mentioned earlier, the proposed MA was fabricated on a highly flexible polyimide film, which can be used in non-planar applications. As shown in Fig. 11a, the absorber was curved and attached to a cylinder with 8 cm radius, and its absorption was then measured. Figure 11b shows absorption spectra for the flat and conformal absorber. It can be observed that the absorptivity of both absorbers is similar. Moreover, peak absorption at the three resonant frequencies was similar before and after bending, which is important in conformal applications.

## Conclusion

An ultra-thin, flexible MA with three absorption peaks is presented in this paper. Compared with previous designs, our proposed absorber is ultrathin with total thickness of 0.4 mm, which is approximately 1/88 the free-space wavelength corresponding to the lower absorption frequency. The proposed triple-band absorber exhibits high absorption up to a 60° angle of incidence (above 86% and 99% for TE and TM polarization, respectively). Meanwhile, the symmetry of the structure ensures absorption is insensitive to changes in polarization. An MA with 20 × 30 unit cells was fabricated and measured for different angles of incidence. The results show that the MA exhibits high absorption at large incident angles. The absorber was fabricated on a flexible polyimide film that can be easily used in the non-planar and conformal applications. The proposed absorber has great potential uses in energy-harvesting and electromagnetic shielding.

## Data Availability

All data are fully available without restriction.

## Abbreviations

MA: Metamaterial absorber
EM: Electromagnetic
SRR: Split ring resonator
MRR: Modified ring resonator
FDTD: Finite-difference time-domain
